# Factors associated with severe disease from malaria, pneumonia and diarrhea among children in rural Tanzania – A hospital-based cross-sectional study

**DOI:** 10.1186/1471-2334-12-219

**Published:** 2012-09-14

**Authors:** Catherine Kahabuka, Gunnar Kvåle, Sven Gudmund Hinderaker

**Affiliations:** 1 Centre for International Health, Faculty of Medicine and Dentistry, University of Bergen, Bergen, Norway; 2 Centre for International Health, Overlege Danielsens Hus, Årstadveien 21, Bergen, 5009, Norway

**Keywords:** Severe, Non-severe, Malaria, Pneumonia, Diarrhea, Dehydration

## Abstract

**Background:**

Mild cases of malaria, pneumonia and diarrhea are readily treatable with complete recovery and with inexpensive and widely available first-line drugs. However, treatment is complicated and expensive, and mortality is higher when children present to the hospital with severe forms of these illnesses. We studied how care seeking behaviours and other factors contributed to severity of malaria, pneumonia and diarrhoea among children less than five years in rural Tanzania.

**Methods:**

We interviewed consecutive care-takers of children diagnosed with malaria, pneumonia and/or diarrhea at Korogwe and Muheza district hospitals, in north-eastern Tanzania, between July 2009 and January 2010, and compared characteristics of children presenting with severe and those with non-severe disease.

**Results:**

A total of 293 children with severe and 190 with non-severe disease were studied. We found persistent associations between severity of disease and caretaker’s lack of formal education (OR 6.6; 95% confidence interval (CI) 2.7-15.8) compared to those with post-primary education, middle compared to high socio-economic status (OR 1.9; 95% CI 1.2-3.2), having 4 or more children compared to having one child (OR 2.5; 95% CI 1.4-4.5), having utilized a nearer primary health care (PHC) facility for the same illness compared to having not (OR 5.2; 95% CI 3.0-9.1), and having purchased the first treatment other than paracetamol from local or drug shops compared to when the treatment was obtained from the public hospitals for the first time (OR 3.2; 95% CI 1.9-5.2). The old officially abandoned first line anti-malaria drug Sulfadoxin-pyrimethamine (SP) was found to still be in use for the treatment of malaria and was significantly associated with childrens’ presentation to the hospital with severe malaria (OR 12.5; 95% CI 1.6-108.0).

**Conclusions:**

Our results indicate that caretakers with no formal education, with lower SES and with many children can be target groups for interventions in order to further reduce child mortality from treatable illnesses. Furthermore, the quality of the available drug shops and PHC facilities need to be closely monitored.

## Background

Mild cases of malaria, pneumonia and diarrhea are treatable with complete recovery from the widely available and inexpensive first line drugs. However, the three diseases still account for the majority of child deaths among children between one month and five years of age in sub-Saharan Africa, including Tanzania [1,2]. Mortality is higher when children present to the hospital with severe compared to mild forms of these illnesses [3,4]. In one of the studies, conducted among children admitted to a Pediatric Emergency Room in India, it was found that the case fatality rates for non-severe, severe and very severe pneumonia were 0%, 8.7% and 47.0% respectively [4].

There have been many efforts to prevent infections in children through vaccination programmes and other community interventions, some with success. One example is the reduction of malaria incidence following the wide distribution of insecticide-treated mosquito nets [5,6]. However, primary prevention of infectious diseases is still difficult to achieve particularly for children in poor families who are continuously exposed to health risks and other hazards typical in poor communities. In addition, many children from poor families are undernourished, making them less resistant to infections. This makes early disease detection and timely management crucial in preventing deaths of these children from treatable illnesses.

There has been an unacceptable widening gap in child mortality between rich and poor countries as well as between wealthy and poor children within many countries (9). Within countries child mortality is often higher in rural areas, and among the poor and less educated families [7,8]. These inequities are compounded by the reduced access to available preventive and curative interventions by children from poor families. Public subsidies for health services frequently benefit rich people more than the poor [8]. Health interventions targeting children from poor families may significantly contribute to further reduction in child mortality from treatable illnesses.

We wanted to know what characterizes children who are more likely to develop severe disease from malaria, pneumonia and diarrhoea. Hence, we conducted a hospital based cross-sectional survey in two predominantly rural districts of Tanzania, and compared children presenting at two district hospitals with severe from those presenting with mild forms of malaria, pneumonia and diarrhoea. Our main objective was to assess determinants of severe disease, particularly health care seeking factors, among under-five children in the study area.

## Methods

### Study area

The study was conducted at Korogwe and Muheza district hospitals of Tanga Region in north-eastern Tanzania. The two hospitals serve as referral hospitals in the two districts. Korogwe district is located about 100 km inland from Tanga. Based on the 2002 Census and a population growth rate of 1.2% per year [9], the projected population in 2009 was 282,901. The district is served by 47 dispensaries, four health centres, one district hospital and two church owned hospitals. Muheza district is around 25 km from the city of Tanga and is about 70 km from Korogwe. Based on 2002 census and a population growth rate of 1.4%, it had a projected population of 306,862 in 2009 [9], which is served by 54 dispensaries, four health centres, and one district hospital. The two districts are predominantly rural and the people are mainly subsistence farmers of maize, cassava, oranges, coconut, rice and banana. Malaria is the leading cause of admissions and deaths among under-five children in both districts [10,11].

### Study design and sample size

This was a cross-sectional hospital based survey among care-takers who brought their sick children at Korogwe and Muheza district hospitals. The target population was care-takers of children between 1 month and 5 years, with a diagnosis of malaria, pneumonia or diarrhoea seen at the outpatient clinics in the two hospitals. In order to detect a difference of 15% in risk of having severe disease, assuming that the risk factor is present in 35% severe and 20% non-severe cases, granted 80% statistical power and a 95% confidence level, we needed at least 296 cases.

### Data collection

Data was collected between July 2009 and January 2010. We identified all sick children presenting with symptoms suggestive of our diseases of interest to the outpatient department between 09am and 02pm i.e. fever, cough, difficult or fast breathing, diarrhoea and vomiting. These were reviewed and assigned diagnosis as per WHO guidelines [12] by the principal investigator or a trained clinical officer. Children with a diagnosis of malaria, pneumonia or diarrhoea were included in the study. While the diagnosis of pneumonia and diarrhoea was reached clinically, malaria diagnosis was confirmed with a rapid malaria diagnostic test (Paracheck®).

Interviews were conducted by trained clinical officers after obtaining a written informed consent. Children who did not need admission were interviewed in a room located near the outpatient department while caretakers of children who were admitted to the hospital were interviewed later within their respective wards, after the child had received initial treatment. The information collected included care-seeking information with all treatments received and places they were obtained from, availability and use of primary care facilities and referral information. Other information collected included questions about indicators of socio-economic status such as household characteristics, composition and assets. All children were also assessed for weight and mid-upper arm circumference (measured at the mid-point between the tip of the shoulder and the tip of the elbow). Caretakers of fifteen severely ill children who died before the interviews could be conducted were not included.

### Ethical consideration

Ethical approval was obtained from the National Institute for Medical Research in Tanzania. Prior to conducting the interviews, informed written consent was obtained from all caretakers for their participation and participation of their children, and none refused. All study procedures were conducted with caution not to interfere with the patients’ ordinary consultations and only after the child had initiated all the necessary treatment.

### Data analysis

Data was double-entered and validated using Epidata version 3.1 and SPSS version 18 was used for analysis. Severe disease included all children with one or more of the following diagnoses: severe malaria, severe and very severe pneumonia, and acute watery diarrhoea (AWD) with some and with severe dehydration. Mild disease included non-severe malaria, mild pneumonia and AWD with no dehydration. We performed bivariate analysis to examine associations between child’s disease severity status and potential predictors. Multivariate analyses were used to determine predictors that remained associated with severe disease when adjusted for other factors. The variables selected for the multivariate model were either significant (p-value <0.05) in the bivariate analysis or shown to be significant in previously published studies.

## Results

**Figure 1:**
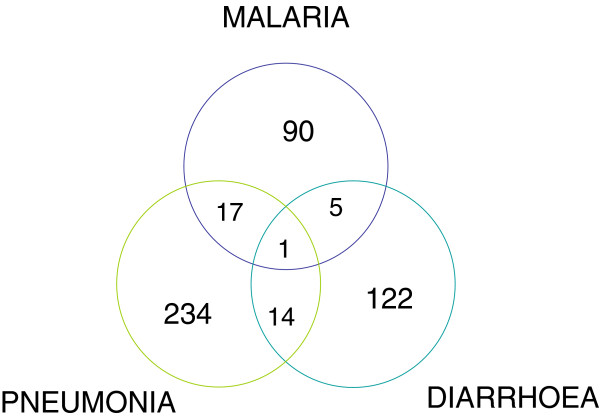
**Distribution of children in relation to the three diseases.** SEVERE CASES: Malaria = 51, Pneumonia = 150, Diarrhoea = 102.

We completed interviews with 560 caretakers of children with a history of symptoms suggestive of malaria, pneumonia or diarrhoea. Out these children, 483 qualified for one or more of above diagnoses and 293 were classified as severe cases. Thirty seven children had more than one diagnosis and if one or more of the diagnoses were severe then the child was categorized as a severe case. Figures 1 summarises the proportion of children with the three diseases together and their severity classification.

The number of study participants enrolled from each district was fairly equal; 258 (53.7%) from Korogwe versus 225 (46.3%) from Muheza. Children were fairly equally distributed by sex and 37% were below one year (Table 1). The majority of caretakers had primary education (75.8%) and were peasants (61.1%). Most caretakers reported having two to three living children and 121 (25.1%) had experienced at least one child death (aged less than five years) in the past.

**Table 1:** Multiple logistic regression of possible background risk factors for severe disease among studied children (n = 483)

**Risk factor for disease severity**	Total cases**	**Severe disease cases (%)**	**OR**	**AOR**
**Child’s Sex**				
Female	230	146 (63.5)	1.3 (0.9-1.8)	1.3 (0.9-2.0)
Male	253	147 (58.1)	ref	ref
**Child’s Age**				
0-11 months	179	116 (64.8)	1.7 (1.1-2.6)*	1.8 (1.1-2.8)*
12-23 months	131	87 (66.4)	1.8 (1.1-2.9)*	1.9 (1.1-3.0)*
24+ months	172	90 (52.3)	ref	ref
**Child’s Weight**				
0-9.9 kg	253	169 (66.8)	1.8 (1.2-2.6)*	1.6 (0.9-3.0)
10.0-14.9 kg	204	109 (53.4)	ref	ref
15.0-20+ kg	22	12 (54.5)	1.0 (0.4-2.5)	1.2 (0.5-3.2)
**Child’s MUAC**				
8.0-13.9 cm	160	113 (70.6)	2.1 (1.3-3.3)*	1.6 (1.0-2.6)*
14.0-14.9 cm	139	80 (57.6)	1.2 (0.7-1.8)	1.0 (0.6-1.7)
15.0-20.0 cm	177	95 (53.7)	ref	ref
**Caretaker’s Education**				
No formal education	78	64 (82.1)	5.9 (2.5-13.9)*	6.6 (2.7-15.8)*
Primary education	366	212 (57.9)	1.8 (0.9-3.5)	2.0 (1.0-4.0)*
Post-primary education	39	17 (43.6)	ref	ref
**Caretaker’s SES**				
Low	174	106 (60.9)	1.6 (1.0-2.7)*	1.5 (0.9-2.6)
Middle	217	142 (65.4)	1.9 (1.2-3.2)*	1.9 (1.2-3.2)*
High	91	45 (49.5)	ref	ref
**No. of own alive children**				
1 child	137	69 (50.4)	ref	ref
2-3 children	240	149 (62.1)	1.6 (1.1-2.5)*	1.7 (1.1-2.7)*
4 or more children	103	74 (71.8)	2.5 (1.5-4.3)*	2.5 (1.4-4.5)*
**Home-hospital travel time**				
0-59 min	164	81 (49.4)	ref	ref
60-119 min	136	79 (58.1)	1.4 (0.9-2.2)	1.4 (0.9-2.3)
120+ min	143	99 (69.2)	2.3 (1.4-3.7)*	2.1 (1.3-3.4)*

OR – Unadjusted odds ratio (from bivariate analysis). AOR- Adjusted for child’s ***Age*** and ***Sex***, and Caretakers ***Education*** and ***socio-economic status (SES).***

* - significant findings.

**- Varying total due to missing cases . MUAC – Mid Upper Arm Circumference.

We used logistic regression to explore any potential associations between participants’ background characteristics and severe disease in our sample of children (Table 1). We found severe disease to be slightly more common among younger compared to older children (64.8% among infants versus 52.3% among children aged two or more years) as well as among children with smaller mid-upper arm circumference (70.6% among children with MUAC between 8.0-13.9 cm compared to 53.7% among those with MUAC of 15 cm and above). Caretakers with no formal education had significantly higher chances of presenting with a severely ill child compared to those with primary and post-primary education (82.1% versus 57.9% and 43.6% respectively). Risk of severe disease was also positively associated with lower socioeconomic status, increasing number of caretakers’ own alive children and increasing travel time from home to the district (study) hospital (Table 1).

Almost all care-takers (442/483; 91.5%) had taken some action within the first 24 hours after recognizing the first child’s symptom. Fever was most frequently reported to be among the first symptoms (61.7%), followed by cough (44.5%). Diarrhea and vomiting was reported to be among the first symptoms in 23.4% and 21.6% of children respectively. Convulsions was experienced by 45 (9.3%) children but was only mentioned as the first symptom in 15 (3.1%) children.

The first option of care for the majority (50.5%) was giving treatment purchased from local and drug shops, mainly paracetamol. A few (15.3%) had given treatments available at home, commonly from previous consultations. Only 104 (21.5%) caretakers reported to have taken their children to the public hospitals within the first 24 hours of onset of symptoms.

Medications given at home were commonly leftovers of previous consultations but were also reported to be purchased from local shops. These included, apart from paracetamol and oral rehydration fluid, tablets like metronidazole, zinc, salbutamol, cotrimoxazole, and syrups like quinine and amoxicillin. Drug shops had more medications and also the specially-packed first line anti-malaria drug (ALu®) that was only to be obtained from public facilities at the time of data collection.

The majority of caretakers (60.5%) who reported not to utilize their nearer PHC facilities had obtained their first treatment other than paracetamol at the study hospital, most of them (77.6%) within 1–2 days of onset of child’s symptoms. Likewise, majority of caretakers (84.5%) who reported having utilized their nearer PHC facilities had also obtained their first treatment, other than paracetamol, from the public hospitals (PHC facility visited first or study hospital). However, only 24.8% had received this treatment within 1–2 days after the child got symptoms; 35.8% had received it on the 3^rd^ or 4th day while the remaining 39.4% got it on the 5^th^ day or later.

Severe disease was more common among children with longer symptoms duration and among children who had utilized Primary Health Care (PHC) facilities for the same illness (Table 2). Children who had received treatments other than paracetamol for the same illness before coming to the district hospital had a higher probability of presenting with severe disease as compared to those who had received paracetamol only (67.9% versus 51.8%). Severe disease was also more common among children who had obtained the first treatment other than paracetamol from local sources or drug shops.

**Table 2:** Multiple logistic regression of potential care-seeking risk factors for severe disease among studied children (n = 483)

**Risk factor for disease severity**	Total number of cases **	**Cases with severe disease (%)**	**OR**	**AOR**
**Symptoms duration**				
1-2 days	246	121 (49.2)	ref	ref
3-4 days	137	102 (74.5)	3.0 (1.9-4.8)*	2.9 (1.8-4.7)*
5 or more days	100	70 (70.0)	2.4 (1.5-4.0)*	2.1 (1.3-3.6)*
**First Rx PCM alone for 24 hrs or more**				
No	299	203 (67.9%)	2.0 (1.3-2.9)*	1.8 (1.2-2.7)*
Yes	170	88 (51.8%)	ref	ref
**Source of first Rx other than PCM**				
Home/local or drug shop	123	95 (77.2)	2.9 (1.8-4.7)*	3.2 (1.9-5.2)*
Private hospital	25	16 (64.0)	1.5 (0.7-3.5)	1.9 (0.8-4.8)
Public hospital	330	178 (53.9)	ref	ref
**Local herbs use**				
No	433	259 (59.8)	ref	ref
Yes	50	34 (68.0)	1.4 (0.8-2.7)	1.4 (0.7-2.8)
**Use of nearer PHC facility**				
No	191	93 (48.7)	ref	ref
Yes	132	110 (83.3)	5.3 (3.1-9.0)*	5.2 (3.0-9.1)*

OR – Unadjusted odds ratio (from bivariate analysis). AOR - Adjusted for child’s ***Age*** and ***Sex***, and Caretakers ***Education*** and ***socio-economic status (SES).***

* - significant findings.

**- Varying total due to missing cases.

PCM = paracetamol. Rx = treatment. PHC = primary health care.

Table 3 below depicts results from multiple logistic regression analysis to compare risk factors for disease severity in individual diseases. The findings indicate that caretaker’s number of living children was strongly associated with malaria severity while it was weakly associated with diarrhoea severity and not with pneumonia severity. On the other hand, caretaker’s SES and travel time to district hospital was associated with severity of pneumonia and diarrhoea but not with severity of malaria. Use of a nearer PHC facility was the only factor that was found to be significantly associated with disease severity from all the three diseases.

**Table 3:** Comparison of potential background risk factors for disease severity among individual diseases (n = 483)

**Risk factor for disease severity**	**AOR**	**AOR**	**AOR**	**AOR**
	**(All cases)**	**(Malaria cases)**	**(Pneumonia cases)**	**(Diarrhoea cases)**
**Caretaker’s Education**				
No formal education	6.6 (2.7-15.8)*		1.8 (0.7-4.7)	3.5 (0.6-21.4)
Primary education	2.0 (1.0-4.0)*	§	1.7 (0.7-3.9)	2.2 (0.4-10.6)
Post-primary education	ref		ref	ref
**Caretaker’s SES**				
Low	1.5 (0.9-2.6)	1.2 (0.4-3.7)	1.8 (0.9-3.8)	1.7 (0.6-4.8)
Middle	1.9 (1.2-3.2)*	1.0 (0.4-2.8)	2.2 (1.1-4.2)*	1.2 (0.4-3.5)
High	ref	ref	ref	ref
**No. of own alive children**				
1 child	ref	ref	ref	ref
2-3 children	1.7 (1.1-2.7)*	3.2 (1.0-10.4)*	0.7 (0.4-1.4)	2.0 (0.8-4.9)
4 or more children	2.5 (1.4-4.5)*	8.5 (2.3-30.6)*	1.5 (0.6-3.3)	1.1 (0.4-3.2)
**Home-hospital travel time**				
0-59 min	ref	ref	ref	ref
60-119 min	1.4 (0.9-2.3)	0.4 (0.1-1.5)	1.5 (0.8-2.7)	2.1 (0.7-6.2)
120+ min	2.1 (1.3-3.4)*	0.9 (0.3-2.9)	1.6 (0.9-3.1)	3.9 (1.4-10.9)*
**First Rx PCM alone for 24 hrs or more**				
No	1.8 (1.2-2.7)*	0.9 (0.4-2.2)	1.8 (1.1-3.3)*	1.7 (0.7-4.4)
Yes	ref	ref	ref	ref
**Source of first Rx other than PCM**				
Home/local or drug shop	3.2 (1.9-5.2)*		2.4 (1.3-4.5)*	1.2 (0.5-2.8)
Private hospital	1.9 (0.8-4.8)	§	1.7 (0.5-5.5)	0.3 (0.1-1.5)
Public hospital	ref		ref	ref
**Use of nearer PHC facility**				
No	ref	ref	ref	ref
Yes	5.2 (3.0-9.1)*	4.2 (1.7-10.5)*	5.2 (2.3-11.9)*	4.4 (1.2-15.8)*

AOR- Adjusted for child’s *Age* and *Sex*, and Caretakers *Education* and *socio-economic status (SES*).

§ - Numbers too small to enable any comparison across groups.

* - significant findings.

PCM = paracetamol. Rx = treatment. PHC = primary health care.

SP (sulphadoxine-pyrimethamine or Fansidar®, formerly first line anti-malaria drug) was reported by 19 caretakers as the only drug provided to their children for the treatment of malaria (suspected or confirmed by tests). Five caretakers reported receiving this drug from public facilities, 6 from drug shops, 7 from private hospitals and 1 from unknown source. Eight out of nine children who received SP and who were confirmed having malaria by tests at the district hospital, had presented with severe malaria, two of them died. SP use for confirmed malaria treatment was highly associated with severe malaria; 89% of children treated with SP had presented with severe malaria compared to only 42% among those who had not received SP (P < 0.01).

## Discussion

In this study from rural Tanzania, we found that children had a higher probability of presenting at the district hospital with severe disease if they had utilized PHC facilities for the same illness, had obtained the first treatment other than paracetamol from local sources and drug shops, and if they had received SP as the only treatment for malaria. Presenting with severe disease was also associated with caretakers’ low level of education and poor nutritional status of children.

An increased risk of severe disease among children of caretakers who reported having utilized their nearer PHC facilities for the same illness might have resulted from selection bias. Caretakers of children who improved after having utilized PHC facilities obviously needed not to come to the district hospital. In addition to that, caretakers of children with severe disease who do not recover after utilizing PHC facilities might be more often referred to and seen at the district hospitals than those with severe disease who do not seek care at PHC facilities. However it is worth noting that, the majority of caretakers who reported having utilized their nearer PHC facilities had obtained the first treatment other than paracetanol much later (on the third day or later) as compared to those who did not utilize them. Delays in treatment initiation among PHC users could be explained by the reported frequent shortages of drugs at these facilities [13], also reported in the study area [14]. Unavailability of drugs at PHC facilities may result in delays of several days, from the time drugs are prescribed until when caretakers are able to purchase them from the drug shops. This finding needs further investigation.

The preference for drugs from local shops as the first option of care shown in this study is not a new finding [15-17]. The positive association between obtaining the first treatment other than paracetamol from these shops and severe disease in children is worth noting. Drug shops in Tanzania are very poorly regulated and some of them sell unregistered and sometimes expired drugs [18]. On the other hand, the reported use of drugs kept at home from previous consultations needs further investigation as this practice could be associated with treatment failures resulting from the use of expired drugs. Oral suspensions of some of the commonly prescribed syrups like amoxicillin and cotrimoxazole should be used only for a maximum of 7 days and then discarded. Caretakers might be using these syrups for extended periods of time.

A quarter of our study participants had experienced at least one child death in the past. This depressing finding calls for urgent measures to improve the situation. The high number of deaths among children may be linked to some of the reported unexpected practices at PHC facilities in the study area [19], including the ongoing provision of SP for malaria treatment in children. Malaria is still the number one killer of children below five years in Africa [20], as well as in Tanzania [21]. Resistance of malaria parasites to SP was commonly reported in many parts of Africa as well as Tanzania between 1999–2000 [22]. In a study conducted in 2000 in Muheza district (one of our study districts), it was found that 45% of the patients treated with SP failed to clear their parasitemias to below patency levels on day 7 [23]. In 2004, the World Health Organization (WHO) recommended that all countries revise their malaria treatment policies and opt for a combination treatment, preferably an artemisinin-based combination therapy (ACT) [24]. Hence, Tanzania changed its first-line treatment for malaria from sulphadoxine-pyrimethamine (SP) to artemether-lumefantrine (ALu®) in 2005 [25]. With funding from the Global Fund, Tanzania began, in December 2006, providing ACTs through public and mission health facilities free of charge to all children under five years and at a subsidized price for the rest of the population [26]. Surprisingly, five out of nineteen children who received SP as the only treatment for malaria reported having received it from public hospitals.

At the time of this study (2009/2010), subsidized ALu® was not yet available outside public facilities in the study area [27]. Hence we predict that SP use for malaria treatment at the community level could be even higher than we have reported in this hospital – based study, and even much higher among caretakers who do not utilize public health facilities (not represented in this study). Further research is needed to ascertain the extent to which this drug is still in use for malaria treatment in children, as this drug was highly associated with severe malaria and death in our sample of children.

Availability of ACT products in the private sector has generally been limited to registered pharmacies in urban areas where the price of a course of therapy is around 8–10 U.S. dollars [28]. The new treatment is well beyond the reach of individuals living in rural and peri-urban communities who need them the most. This has left millions of Tanzanians in rural areas, including the study area, to rely on public sector facilities for access to the recommended first-line treatment for malaria. If they seek treatment from other sources they may end up receiving suboptimal therapies, such as SP, which is more affordable and widely available. However, a few caretakers in our study had reported to occasionally having bought the specially packed subsidized ALu® for public facilities in their nearby local drug shops when these drugs were out of stock at their nearby PHC facilities. This finding raises an alarm.

There have been efforts to make subsidized ACTs available through Accredited Drug Dispensing Outlets (ADDOs) after realizing an important role played by this sector in provision of malaria treatment [28]. In 2007, using funding from the President's Malaria Initiative (PMI) and technical support from Rational Pharmaceutical Management Plus (RPM Plus) Program, Tanzania Food and Drugs Authority (TFDA) and National Malaria Control Programme (NMCP) began a pilot program to make subsidized ACTs available through ADDOs in 10 districts of Morogoro and Ruvuma regions [28]. The program resulted in a gradual increase in the number of malaria patients treated with ACTs, from 3% of all antimalarials sold in July 2007 to 26% in June 2008. At the same time, the use of non-ACT antimalarials like SP declined. Currently, the government of Tanzania is trying to expand the provision of the subsidized ALu® through ADDOs countrywide.

In line with our findings, several previous studies had established severe disease to be more common among younger children and children of caretakers with lower level of education and SES [3,29,30]. In one hospital-based study conducted in southern Kerala, India, they found that severe pneumonia was less frequent in children over 2 years [30]. Likewise in our study severe disease was found to be more common in children less than two years as compared to those aged two years and above. In the Indian study above [30], paternal and maternal education of up to middle school and above was found to be protective against development of severe pneumonia. Similarly, our study showed an inverse association between severe disease and caretakers’ level of education. One community-based study in rural southern Tanzania found that it was more likely for caretakers with higher SES to seek care from an appropriate provider as compared to those with lower SES [8]. In the same study, the frequency of antibiotic use for probable pneumonia among children with the lowest SES was less than half of those with the highest SES, and children in the lowest SES group were half as likely to have been given antimalarials as those in the highest SES category.

The observed positive association between severe disease and more number of children may be due to confounding; explained by the fact that caretakers with low education and SES also tend to have more children. However, the association persisted even after controlling for education and SES. This finding needs more investigation as it could provide another target group for the fight towards child mortality from treatable illnesses.

### Methodological strengths and limitations

The present study has reported factors associated with severe disease from the three main childhood killers (malaria, pneumonia and diarrhea), thus providing more areas for further intervention towards child mortality from treatable illnesses. This study inquired care-seeking information related to the current child illness. By using this approach, we believe we minimized recall bias as we were able to pick up easily forgettable care-seeking information which community based studies may miss, often relying on past illness episodes of preceding weeks.

Our study was hospital-based and was conducted at district referral hospitals because this was the most convenient way to study factors associated with severe disease. To avoid the selection bias, the study should ideally have been community-based, including all children with the three diseases in the area. However, it would be very difficult to identify and include a high enough number of severely ill children at the community level. Hence, we acknowledge that we missed mild and severe cases that used other options of care and did not come to the district hospitals. However, biased associations would only arise if an interaction is present between severity of disease and potential predictors in relation to the frequency of seeking hospital care. As discussed above, this might be the case when attending PHC facilities is studied as a risk factor, but seems less likely to influence the findings of the other predictors. Furthermore, our findings are similar to findings from previously community-based studies on risk factors for early childhood deaths [8,31,32].

Thirty seven children had more than one diagnoses; one child had all three diagnoses. We do not think this influenced our findings significantly as the number of children with more than one severe disease was very small. The fact that we did not include caretakers of fifteen children who died before the interviews could be conducted could have biased our findings towards milder disease. However since the number is too small we think the bias should be minimal.

## Conclusions

Our study has identified some factors that are associated with severity of disease from malaria, pneumonia and diarrhea. We argue that health interventions addressing the factors and risk groups identified in this study would be important for further reduction of child mortality from treatable illnesses. The findings also indicate the strong need for district health systems strengthening, including strict regulations and close quality monitoring of the available PHC facilities and drug shops. Further studies are needed for providing more evidence of the ways and extent by which low quality of services at the existing PHC facilities and drug shops contribute to high mortality among children in developing countries.

## Abbreviations

SP: Sulfadoxine-pyrimethamine (old-first line antimalarial drug same as Fansider®); ALu®: Artemether Lumefantrine ( current first-line antimalaria drug); AWD: Acute watery diarrhoea; PCM: Paracetamol; PHC: Primary Health Care; WHO: World Health Organization; ADDOs: Accredited Drug Dispensing Outlets.

## Competing interests

The authors declare that they have no competing interests.

## Author’s contributions

CK planned and wrote the protocol, collected and analysed data, and drafted the manuscript. GK had substantial contribution to the protocol development as well as data analysis and writing the manuscript. SGH supervised data analysis and contributed in writing the manuscript. All authors have read and approved the final version.

## Pre-publication history

The pre-publication history for this paper can be accessed here:

http://www.biomedcentral.com/1471-2334/12/219/prepub
